# Progesterone-Only Contraceptive-Induced Ischemic Colitis

**DOI:** 10.14309/crj.0000000000001313

**Published:** 2024-04-01

**Authors:** Medha Rajamanuri, Meher Garg, Harris Siddiqui, Sreeram Pannala

**Affiliations:** 1 Southern Illinois University-School of Medicine, Springfield, IL; 2 Springfield High School, Springfield, IL; 3 Staten Island University Hospital, Staten Island, NY

**Keywords:** Ischemic colitis, Progesterone-only contraceptives, Contraceptive side effects, Bowel ischemia, Hormonal contraceptives

## Abstract

Ischemic colitis (IC) occurs when there is a temporary lack of adequate blood supply to the intestines, particularly in vulnerable areas such as the splenic flexure and sigmoid colon, which lack sufficient collateral blood vessels. Although primarily seen in older individuals with atherosclerotic risk factors, IC can also be triggered by hormonal contraceptives in young women. Although estrogen-containing oral contraceptives are known to increase thromboembolic risk, the impact of progesterone is not well understood. We report a unique case of biopsy-confirmed IC in a previously healthy 30-year-old woman who presented with abdominal pain and bloody diarrhea 2 months after initiation of progesterone-only oral contraceptive. This occurrence, although rare, underscores the need for further research on the impact of progesterone on coagulation, especially concerning mesenteric arteries.

## INTRODUCTION

Colonic ischemia is the most frequent form of intestinal ischemia with an annual incidence of approximately 1.6 cases per 100,000 individuals, rising to 18 cases per 100,000 among hospitalized patients.^1^ Although older, hospitalized patients with atherosclerotic risk factors are typically affected, it is rare in otherwise healthy young people. Young women may experience ischemic colitis (IC) because of hormonal therapy's prothrombotic effects.^2^ Users of combined oral contraceptives undergo several blood protein changes, such as increased levels of factors II, VII, VIII, and X, along with fibrinogen. Furthermore, there is a decrease in antithrombin and protein S levels. Combined oral contraceptive users also develop resistance to activated protein C because of the enhanced synthesis of factor VII, factor X, and fibrinogen during the initial hepatic metabolism of oral estrogen.^3^ Estrogen-containing oral contraceptives are known to cause venous thromboembolic events, but the impact of progesterone is not well understood.^4^ We present the second documented instance in literature and first biopsy-proven IC because of progesterone-only contraceptive use.

## CASE REPORT

**Figure 1.**
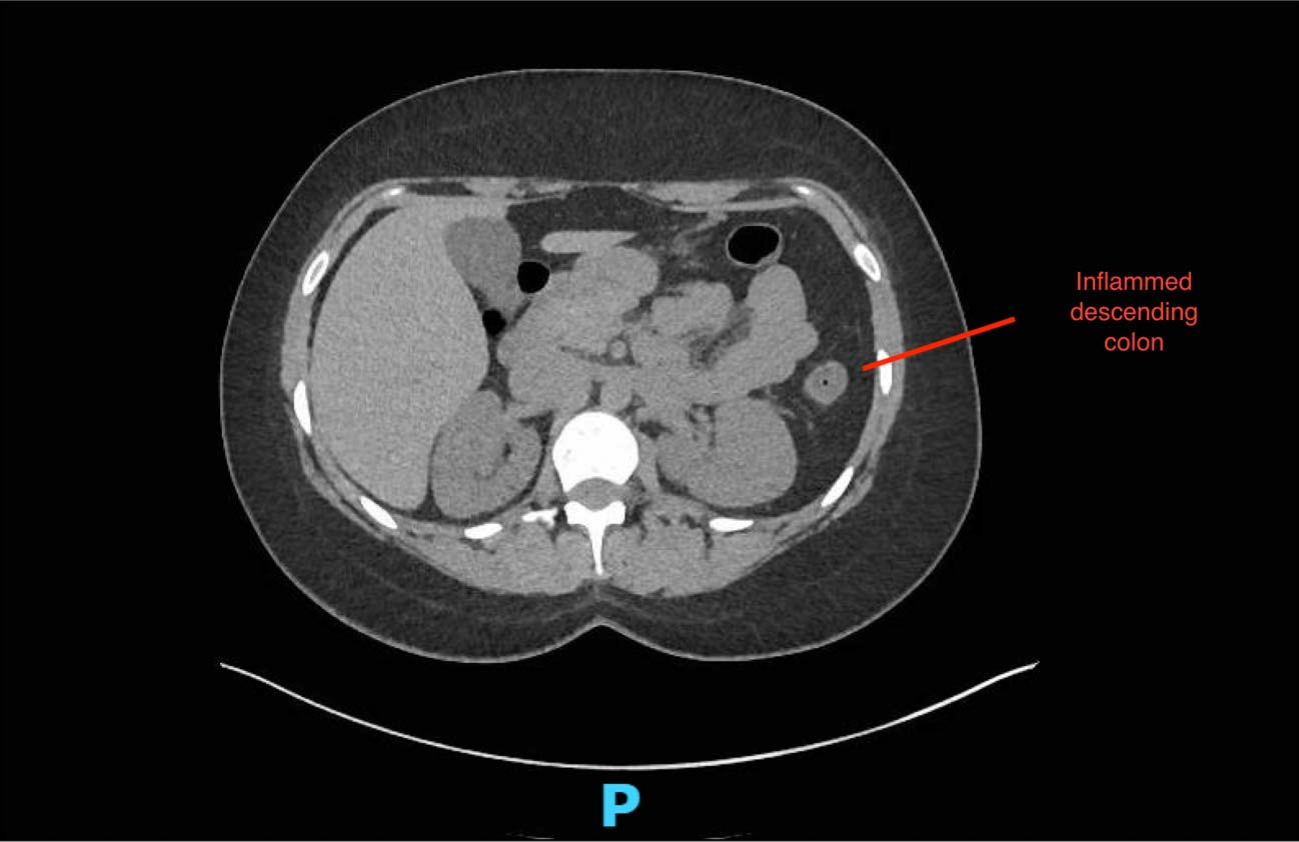
Mild wall thickening and adjacent inflammatory stranding are observed, primarily affecting the mid-distal descending colon, indicative of colitis-associated inflammatory changes.

**Figure 2.**
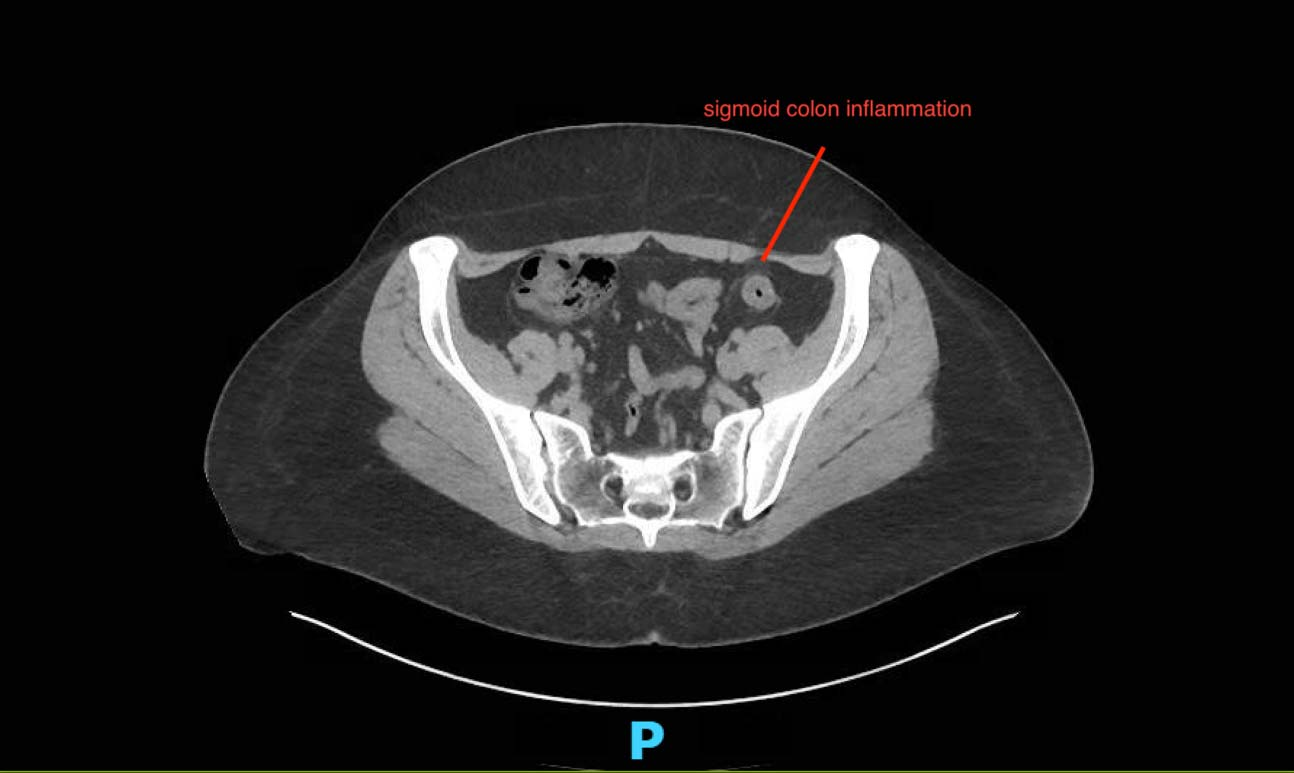
The image reveals mild wall thickening and adjacent inflammatory stranding, consistent with colitis, affecting the distal descending colon and extending into the sigmoid colon.

**Figure 3.**
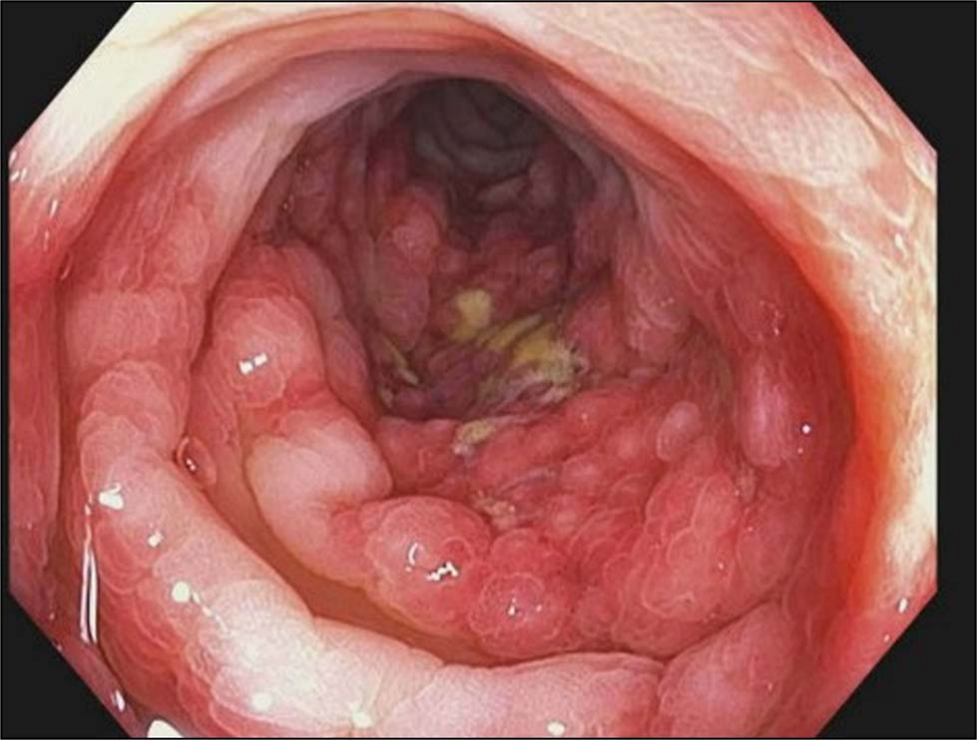
Image depicts edematous and delicate mucosa with segmental erythema and scattered erosions observed in the mid-distal descending colon.

**Figure 4.**
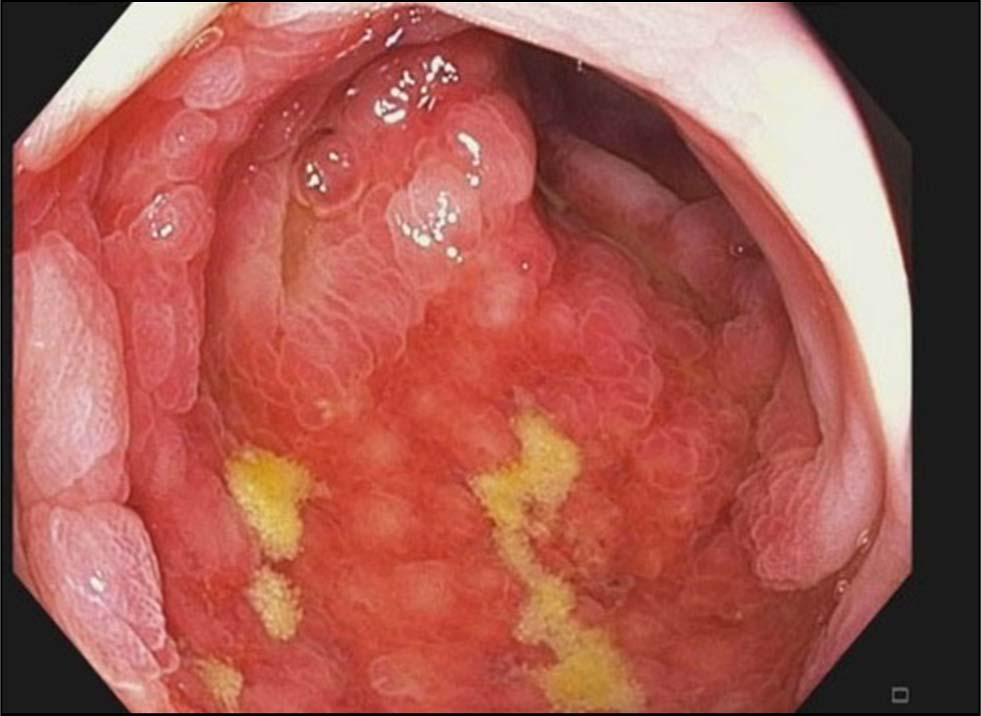
Notable in the image is a region of significantly congested mucosa located in the distal descending colon/proximal sigmoid colon (watershed area), characterized by erythema and ulcerations, suggesting localized tissue damage and inflammation.

**Figure 5.**
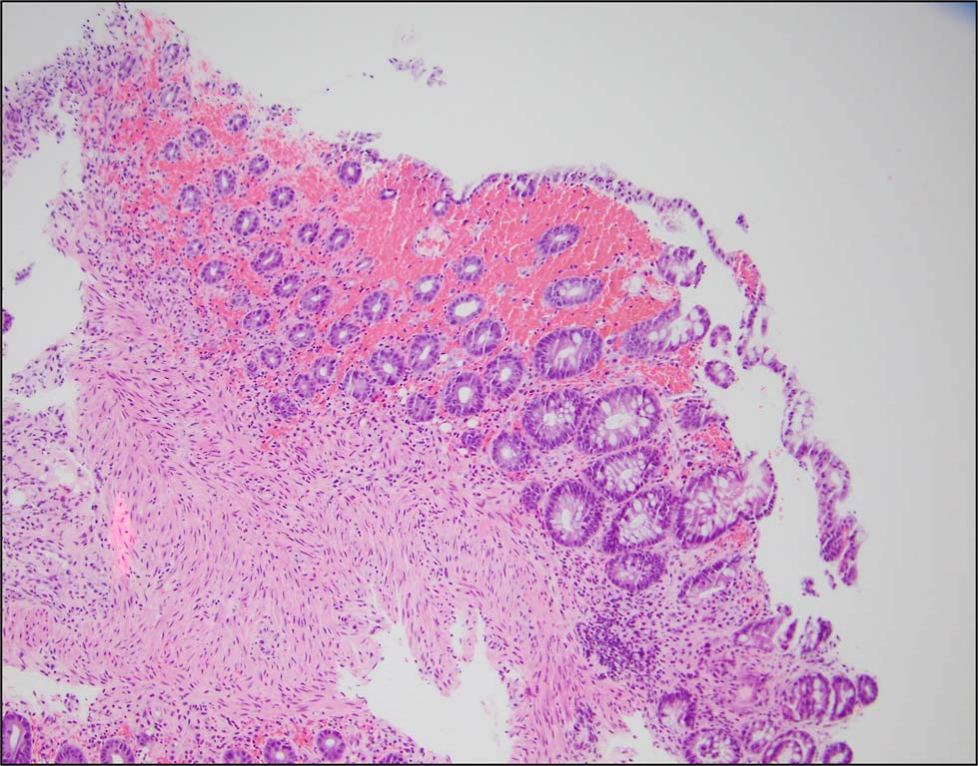
Histopathological examination of the inflamed colon shows ischemic changes, including edema, hemorrhage, and mild crypt architectural distortion.

A 30-year-old white woman with no significant medical history presented to the emergency department with a 3-week history of worsening crampy abdominal pain, nausea, and bloody diarrhea. She denied any history of smoking or alcohol usage. Her vital signs on admission were stable with a blood pressure of 104/64, heart rate of 114, and her body mass index was 32.3. Abdominal examination was positive for normal bowel sounds and diffuse tenderness to palpation but was negative for guarding or rigidity. Her only medication was progesterone-only contraceptives for the past 2 months. She had a normal complete blood count, comprehensive metabolic panel, and coagulation panel, and C-reactive protein was 4.8. Stool studies were negative for cultures and *Clostridioides difficile* toxins. Her fecal calprotectin was elevated at 2006 mcg/g (10–50 mcg/g). A computed tomography scan of the abdomen and pelvis with contrast showed colitis involving mid and descending colon, characterized by mild colonic wall thickening and adjacent inflammatory changes (Figures 1 and 2). No perforation or abscess was noted. She was treated with intravenous fluids, antiemetics, and pain medicines. She received a dose of intravenous ciprofloxacin and metronidazole for possible infectious colitis before a gastroenterology consultation for possibility of colonoscopy. Colonoscopy was significant for segmental erythema and ulceration involving descending and sigmoid colon (Figures 3 and 4). Ascending colon, transverse colon, and rectum were normal-appearing. Histopathology showed evidence of IC with subepithelial edema and hemorrhage and no signs of granulomas or dysplasia (Figure 5). She responded to conservative management with intravenous fluids, antiemetics, and was asked to discontinue progesterone-only contraceptive pill on discharge. Her symptoms improved over a period of 2 weeks after discharge while off of her progesterone-only contraceptive. On a 3-month outpatient visit, she denies any recurrence of symptoms.

## DISCUSSION

IC arises from transient compromise of intestinal vascular supply because of metabolic demands. The colon primarily relies on the superior and inferior mesenteric arteries, interconnected through arcades at the splenic flexure and sigmoid colon. These vulnerable watershed areas, lacking sufficient collateral supply, are susceptible to ischemic insults. Hallmark presentations of IC include crampy abdominal pain, diarrhea, and rectal bleeding. Some patients may complain of upper gastrointestinal symptoms such as nausea and vomiting. Examination is usually positive for abdominal tenderness with possible peritoneal signs especially in severe cases.

The differential diagnosis is broad and not limited to infectious colitis, inflammatory bowel disease, pseudomembranous colitis, diverticulitis, and colon cancer. Early colonoscopy is considered the gold-standard diagnostic tool and can help differentiate between different causes of colonic inflammation. Histopathology usually shows hemorrhage, distorted crypts, edema, and inflammatory infiltration.^5^ Distinguishing features such as segmental involvement, rectal sparing, and rapid resolution help differentiate IC from inflammatory bowel disease.^6^ Treatment varies by severity, often resolving spontaneously on addressing underlying causes, as observed in our patient through cessation of the causative drug.^5^

IC predominantly affects young women, who are on hormonal contraceptives, particularly estrogen, being implicated.^2^ Although estrogen dose correlates with venous thromboembolism risk, progesterone's direct correlation remains uncertain.^4^ Hormonal contraceptives heighten coagulation factors, platelet aggregation, and fibrinogen levels, inducing hypercoagulability.^7^ Ongoing research aims to minimize thrombotic risk by formulating oral contraceptive pills with lower estrogen doses. However, case reports highlight reversible colonic ischemia in patients on third-generation hormonal contraceptives.^8^ This case also raises questions about the possibility of hypercoagulability being induced by progesterone use.

Our case presents a unique scenario of biopsy-confirmed IC after the use of a progesterone-only contraceptive, the second documented case as per our literature review. The first case, reported in 1972 by Martin D. Gelfand, involved a multiparous woman experiencing abdominal cramping, bloody stools, and rectosigmoid erythema after initiating Depo-Provera, a progesterone-only contraceptive.^9^ In cases of contraceptive-induced IC, patients typically recover after discontinuing the hormonal contraceptive. Similarly, our patient had complete resolution of her symptoms within a few weeks of ceasing the medication. The mechanism underlying progesterone-only contraceptives triggering ischemic events remains poorly understood, underscoring the need for further research and more vigilance in patients using progesterone-only contraceptives.

In conclusion, our case is the first biopsy-proven case of IC resulting from progesterone-only contraceptive pill. Despite the widespread usage of progesterone-only contraceptives, the occurrence of IC within this patient population remains unrecognized. Although potential patient-related variables may contribute to disease onset in the absence of a known hypercoagulable state, the precise cause remains elusive. Further studies are needed to better comprehend the impact of progesterone on coagulation, particularly concerning the mesenteric vasculature.

## DISCLOSURES

Author contributions: M. Rajamanuri: manuscript writing and is the article guarantor. M. Garg: literature search and input on the discussion. H. Siddiqui: identified the uniqueness of the case and helped with data collection. S. Pannala: manuscript editing.

Financial disclosure: None to report.

Informed consent was obtained for this case report.
